# Polymorphisms in steroidogenesis genes, sex steroid levels, and high myopia in the Taiwanese population

**Published:** 2011-08-25

**Authors:** Zoe Tzu-Yi Chen, I-Jong Wang, Ya-Tang Liao, Yung-Feng Shih, Luke Long-Kuang Lin

**Affiliations:** 1 Department of Ophthalmology, Taipei City Hospital Zhongxing Branch, Taipei, Taiwan R.O.C; 2 College of Medicine, National Taiwan University, Taipei, Taiwan R.O.C; 3 Department of Ophthalmology, National Taiwan University Hospital, Taipei, Taiwan R.O.C; 4 Graduate Institute of Clinical Medical Science, China Medical University, Taichung, Taiwan R.O.C; 5 Genomics Research Center, Academia Sinica, Taiwan R.O.C

## Abstract

**Purpose:**

To evaluate the relationship among single nucleotide polymorphisms (SNPs) in steroidogenesis enzyme genes, serum levels of sex steroids, and high myopia in Taiwanese male and female populations.

**Methods:**

A campus-based sample of 283 cases (145 males and 138 females) with high myopia and 280 controls (144 males and 136 females) with low myopia or emmetropia was studied. Estradiol, progesterone, and testosterone levels were determined using enzyme-linked immunosorbent assay kits. We genotyped six SNPs within five steroidogenesis enzyme genes (17 alpha-hydroxylase/17,20 lyase [*CYP17A1*], 3 beta-hydroxysteroid dehydrogenase [*HSD3B1*], 17 beta-hydroxysteroid dehydrogenase 1 [*HSD17B1*], steroid-5-alpha-reductase, alpha polypeptide 2 *[SRD5A2*], and aromatase [*CYP19A1*]) using polymerase chain reaction–restriction fragment length polymorphism methods. Student’s *t*-tests, χ^2^ tests, logistic regression, multifactor dimensionality reduction (MDR) methods, and ANOVA were used to determine significance.

**Results:**

An MDR analysis corroborated the synergistic genotype association and demonstrated that synergistic interaction between rs6203 (*HSD3B1*), rs10046 (*CYP19A1*), and sex might confer susceptibility to high myopia (p=0.019). In both male and female subjects, levels of testosterone were significantly higher in cases than in controls; in male subjects, the levels of estradiol were significantly higher and those of progesterone were significantly lower in cases (all p-values <0.001). The rs605059 (*HSD17B1*), with sex-gene interaction, showed association with estradiol levels in males (p=0.035) and testosterone levels in females (p=0.027).

**Conclusions:**

Testosterone levels correlate with high myopia, and interaction of steroidogenesis enzyme genes and sex may be a modulating factor in sex hormone metabolism and high-myopia risk.

## Introduction

Myopia is the most common eye disorder in Taiwan and around the world. A refractive error in excess of −6 diopters (D), defined as high myopia, is also termed “pathological myopia” due to its associated potential complications, including cataracts, glaucoma, macular degeneration, and retinal detachment, which can lead to blindness. Many environmental factors [1,2] have been reported to be associated with myopia. Population, family, and twin studies also have provided evidence for a genetic component to high myopia [3-5]. According to a nationwide survey in Taiwan in 2000, girls have a higher prevalence and more severe myopia progression than boys [6]. Several other epidemiological studies [7,8] have also shown similar results. In addition, the female sex was reported to be independently associated with faster myopia progression [9]. Because of sex differences, a hormonal hypothesis has been postulated by a review article examining myopia in opposite-sex twins [10]. In animal models, sex differences were also demonstrated in chick eye growth and experimental myopia [11]. It has also been shown that myopia progresses quickly in childhood, especially around puberty [6-8,12]. These studies have demonstrated that sex may be a modulating factor affecting myopia and have suggested that the differential expression of sex hormones may be as well. Knowledge about sex differences in the progression of myopia and about the pathophysiology underlying those differences may enhance the accuracy and effectiveness of clinical assessment and treatment of myopia. With a remarkable prevalence of high myopia and a homogenous genetic background, the Taiwanese population would be the best candidate for genetic studies to find myopia susceptibility genes, and approaches focusing on high myopia may minimize the impact of environmental confounders.

Thinning of the sclera is a key feature of human myopia development. Modulated by retinoscleral signals, the scleral remodeling mechanism is common [13-16] to different paradigms of myopia induction. It involves the regulation of numerous gene products (mRNAs and proteins) [17-23] in the extracellular matrix, such as collagens, proteoglycans, matrix metalloproteinases (MMPs), and tissue inhibitors of metalloproteinases (TIMMPs). Sex steroids are present in all tissues because they circulate through the blood; however, their effects can be observed only in cells armed with the corresponding receptors [24]. Studies have demonstrated the presence of androgen, estrogen, and/or progesterone receptors in various ocular tissues, such as the lacrimal gland, meibomian gland, lid, conjunctiva, cornea, iris/ciliary body, lens, retina/uvea, retina/choroid, and retinal pigment epithelial cells [24-28]. Nevertheless, both the levels and activities of these receptor proteins are regulated by hormones in the blood, suggesting that the sex hormone axis may play a role in the pathogenesis of certain ocular diseases.

Progesterone and estradiol are the major naturally occurring human hormones in the groups of progestogens and estrogens, respectively. Testosterone, a steroid hormone from the androgen group, is the principal male sex hormone. Steroids have been reported to change the biochemical characteristics of the iris/ciliary body, aqueous outflow pathway, and sclera in the rabbit eye [29]. In humans, estrogen is a modulating factor of the biomechanical properties of the cornea, which may contribute to the development of keratectasia after corneal refractive surgery [30]. Changes in cornea thickness due to fluctuations in estrogen levels are known to occur during the menstrual cycle [31] and pregnancy [32]. Corneal curvature also changes during pregnancy [33]. Besides, steroidogenesis enzymes and sex steroids have been proved to be related to ocular physiology and diseases [24,34-42]. Hormonal balance has been hypothesized to be related to the weakening of the collagenous tissue of the sclera [43]. However, no studies have been published to date regarding the influence of these sex hormones on the sclera or clinical myopia. Further research on this will be helpful to assess and manage myopia.

Sex steroids such as estrogen and dehydroepiandrosterone (DHEA, belonging to the androgen group) have been reported to regulate MMPs. Estrogen is known to upregulate MMP-2 and/or MMP-9 in mouse and human cells [26,44-46]. Depression of MMP-2 activity, on the other hand, has been shown in the rat polycystic ovary (PCO) induced by DHEA [47]. Because MMP-2 upregulation was suggested to take part in scleral remodeling in myopia development [17,23], and because sex hormones might implicate MMP-2 in the pathogenesis of myopia, our hypothesis is that genetic polymorphisms in steroidogenesis enzyme genes may affect circulating sex steroid levels and thus influence the risk of high myopia.

**Figure 1:**
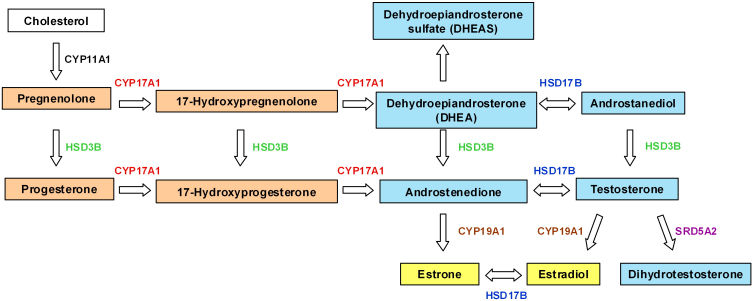
Major pathways in sex steroid biosynthesis (progestogens in orange boxes, estrogens in yellow boxes, and androgens in blue boxes). CYP11A1=cholesterol desmolase, CYP17A1=17 alpha-hydroxylase/17,20 lyase, HSD3B=3 beta-hydroxysteroid dehydrogenase, HSD17B1=17 beta-hydroxysteroid dehydrogenase 1, CYP19A1=aromatase, SRD5A2=steroid-5-alpha-reductase, alpha polypeptide 2.

The progestogens, estrogens, and androgens are the three major classes of sex steroids and are derivatives of cholesterol (Figure 1; progestogens in orange boxes, estrogens in yellow boxes, and androgens in blue boxes). The enzymes involved in the biogenesis of sex hormones include 17 alpha-hydroxylase/17,20 lyase (CYP17A1), 3 beta-hydroxysteroid dehydrogenase (HSD3B1), 17 beta-hydroxysteroid dehydrogenase 1 (HSD17B1), steroid-5-alpha-reductase, alpha polypeptide 2 (SRD5A2), and aromatase (CYP19A1). Several notable nonsynonymous SNPs or common SNPs with functional effects have been reported in these genes, including rs743572 (*CYP17A1*), rs6203 (*HSD3B1*), rs1047303 (*HSD3B1*), rs605059 (*HSD17B1*), rs523349 (*SRD5A2*), and rs10046 (*CYP19A1*) [48-59]. rs743572 (*CYP17A1*) is located in the 5′ UTR and rs10046 (*CYP19A1*) is located in the 3′ UTR, the rest of these SNPs are all in exon areas.

Herein, we report the first case-control study to assess the relationship among genetic variations of steroidogenesis enzymes, sex hormone levels (including progesterone, estradiol, and testosterone), and high myopia.

## Methods

### Ethics statement

The study was approved by the Ethics Committees of the National Taiwan University Hospital, Taipei, Taiwan and followed the tenets of the Declaration of Helsinki. All participants were informed about the nature of the study and signed a written consent form before blood withdrawal.

### Subjects and clinical assessment

A total of 283 unrelated high-myopia cases (145 males and 138 females) with refractive errors in excess of −6 D in both eyes and 280 controls (144 males and 136 females) with refractive errors of −2 D to 1 D in either eye were recruited from the National Taiwan University for this campus-based control study. No participant had ammetropia of more than 2 D or known ocular diseases/insults that could predispose to myopia. All subjects were of Han Chinese origin and were similar in social background. Ocular examination included objective autorefraction and keratometry (KR-8100; Topcon, Tokyo, Japan) and slit-lamp evaluation.

### Measurement of hormone levels

Blood samples were centrifuged at 805× g for 15 min at 4 °C, and serum was separated and stored at −30 °C until analysis. Hormone levels were determined using an enzyme-linked immunosorbent assay (ELISA) kit (Diagnostic Systems Laboratories, San Antonio, TX) according to the manufacturer's instructions. The Steroid ELISA is based on the principle of competitive binding between steroids in the test specimen and steroid-horseradish peroxidase (HRP) conjugate for a constant amount of rabbit anti-steroid (progesterone, estradiol, and testosterone). In the incubation, goat anti-rabbit IgG-coated wells were incubated with standards (25 μl for progesterone and estradiol, 10 μl for testosterone), controls, subject samples (25 μl for progesterone and estradiol, 10 μl for testosterone), 100 μl steroid-HRP conjugate reagent, and 50 μl rabbit anti-steroid hormone reagent at room temperature (18–25 °C) for 90 min. During the incubation, a fixed amount of HRP-labeled steroid hormone competed with the endogenous steroid hormone in the standard, sample, or quality control serum for a fixed number of binding sites of the specific steroid hormone antibody. Thus, the amount of steroid hormone peroxidase conjugate immunologically bound to the well progressively decreased as the concentration of steroid hormone in the specimen increased. Unbound steroid hormone peroxidase conjugate was then removed, and the wells were washed. Next, a solution of H_2_O_2_/TMB reagent (100 μl TMB for progesterone and testosterone, 200 μl H_2_O_2_/TMB only for estradiol) was added and incubated at room temperature for 20 min, resulting in the development of blue color. The color development was stopped with the addition of stop solution (100 μl 1N HCl for progesterone and testosterone, 100 μl 3N HCl only for estradiol) and the absorbance was measured spectrophotometrically at 450 nm. Standards were assayed in duplicate, and unknowns were assayed in triplicate. The results were expressed as ng/ml or pg/ml.

### SNP genotyping

Genomic DNA was extracted from participants’ peripheral leukocytes using an extraction kit (Qiagen GmbH, Hilden, Germany). To analyze polymorphisms in each gene, we used polymerase chain reaction–restriction fragment length polymorphism (PCR-RFLP) methods to directly genotype the polymorphism. The primers for amplifying 50 ng of genomic DNA were as follows: 5′-GCT AGG GTA AGC AGC AAG AG-3′ and 5′-CAA GGT GAA GAT CAG GGT AG-3′ for rs743572 (*CYP17A1*), 5′-GGC TTC CTG CTG GAA ATA GTG A-3′ and 5′-GCA AAC CAC ATG GTC TTT CCT C-3′ for rs6203 (*HSD3B1*), 5′-CAG GCC AAT TTA CAC CTA TCG-3′ and 5′-TCA AAC TAT GTG AAG GAA TGG A-3′ for rs1047303 (*HSD3B1*), 5′-CCT TCC ACC GCT TCT ACC AAT A-3′ and 5′-CAG GGA CCA CAC AGA CCC AG-3′ for rs605059 (*HSD17B1*), 5′-AGG ATT TGG AGC TGC TGA GAG C-3′ and 5′-GAC GGT ACT TCT GGG CCT CTT CTA C-3′ for rs523349 (*SRD5A2*), and 5′-ACT ACT GAT GAG AAA TGC TCC AGA CT-3′ and 5′-GGT TCC TTA TAG GTA CTT TCA GCC A-3′ for rs10046 (*CYP19A1*). The PCR reactions were performed in 25-μl aliquots containing 20 pmole of each primer, 1× reaction buffer, 100 µM deoxynucleotide triphosphates, and 1 unit of Taq polymerase; a PCR system 9700 thermocycler (Applied Biosystems, Foster City, CA) was used for cycling. The temperatures used during PCR were as follows: for rs743572, 95 °C for 2 min, followed by 30 cycles of 95 °C for 1 min, 57 °C for 1 min, and 72 °C for 1 min, with a final extension at 72 °C for 10 min; for rs6203 and rs605059, 95 °C for 2 min, followed by 30 cycles of 95 °C for 30 s, 60 °C for 30 s, and 72 °C for 30 s, with a final extension at 72 °C for 10 min; for rs1047303, 95 °C for 2 min, followed by 40 cycles of 95 °C for 30 s, 50 °C for 30 s, and 72 °C for 30 s, with a final extension at 72 °C for 10 min; and for rs523349 and rs10046, 95 °C for 2 min, followed by 30 cycles of 95 °C for 30 s, 52 °C for 30 s, and 72 °C for 30 s, with a final extension at 72 °C for 10 min. Aliquots of PCR products were digested with the following restriction enzymes and conditions: MspA1I for rs743572 at 37 °C for 3 h, BglI for rs6203 at 37 °C overnight, DrdI for rs1047303 at 37 °C overnight, BtsI for rs605059 at 55 °C overnight, HpyCH4V for rs523349 at 37 °C overnight, and BsrCI for rs10046 at 65 °C overnight, in reaction mixtures containing 10 μl of PCR product, digestion buffer, BSA (BSA), and 5 units of restriction enzyme (New England Biolabs, Ipswich, MA). The digested fragments were visualized on 4% agarose gel with ethidium bromide staining to identify the base pair change. To exclude the possibility of introducing PCR error and miscutting of the restriction enzyme, all steps were repeated twice. For equivocal results, we double-checked with either repeating the experiments or directly sequencing the PCR products using dye terminator chemistry (BigDye Terminator version 3.1 on a model 3730 Genetic Analyzer; Applied Biosystems).

### Statistical analyses

SAS version 9.1 (SAS, Inc., Cary, NC) was used for all statistical comparisons. Comparisons of age, refractive status, and hormone levels between the case and control groups were performed with Student’s *t*-tests. Genotypes were assessed for Hardy–Weinberg equilibrium (HWE), and allele frequencies were compared between cases and controls using χ^2^ tests. Odds ratios (ORs) for specific genotypes and 95% confidence intervals (CIs) were calculated using logistic regression analyses with the wild type genotypes as references. Synergistic analysis was evaluated by pairwise modification of genotypic associations through logistic regression. Global and genotypic-specific p-values were reported. The multifactor dimensionality reduction (MDR) method [60,61] with version 1.1.0 of the open-source MDR software package (Dartmouth Medical School, Hanover, NH) was used to search for the most potential gene-gene interaction models. MDR tests all possible multilocus genotype combinations and reports a combination that provides the best classification [62]. The final models with relatively highest prediction accuracy for single to four-way interactions are selected for cross-validation. This method, which is a nonparametric and model-free approach, combines cross-validation and permutation-testing procedures to minimize false positives caused by multiple testing and has performed well across many genetic simulation scenarios where purely epistatic relationships existed between status and variables without main effects [63]. True-positive models possess estimated testing accuracy of 50% or more cross-validation consistency. ANOVA (ANOVA) including six polymorphisms and sex was performed with the general linear models (GLM) procedure to determine the sources of variation for hormone levels, and F tests computed from the type III sums of squares reported the effect of an independent variable after adjustment for all other independent variables included in the model. Two-way interaction of each polymorphism and sex was also analyzed. Another ANOVA compared between-group hormone levels for different genotypes of steroidogenesis enzyme genes. Data from males and females were also analyzed separately.

## Results

### Subjects

Our study consisted of 283 high-myopia cases (145 males and 138 females; average age 18.8±0.8 years [mean±standard deviation]; average spherical equivalent −8.2±1.7 D for right eyes, −8.0±1.8 D for left eyes) and 280 emmetropia or low-myopia controls (144 males and 136 femals; average age 19.4±2.4 years [mean±standard deviation]; average spherical equivalent −0.8±0.8 D for right eyes, −0.7±0.8 D for left eyes; Table 1).

**Table 1:** Baseline characteristics

	**All subjects**		** **
**Variable**	**Case (n=283)**	**Control (n=280)**	**p-value**
Age (years)	18.8±0.8	19.4±2.4	<0.001
Male (%)	148 (51.0)	147 (51.4)	0.930
SEM of right eye (D)	−8.2±1.7	−0.8±0.8	<0.001
SEM of left eye (D)	−8.0±1.8	−0.7±0.8	<0.001
Progesterone (ng/ml)	3.7±3.8	5.2±6.6	0.008
Estradiol (pg/ml)	34.8±22.1	35.1±30.5	0.895
Testosterone (ng/ml)	4.1±2.8	3.4±2.5	<0.001
**Male subjects**			
** **	**Case (n=145)**	**Control (n=144)**	** **
Age (years)	18.8±1.0	19.2±2.1	0.019
SEM of right eye (D)	−8.3±1.6	−0.9±0.8	<0.001
SEM of left eye (D)	−8.1±1.5	−0.8±0.8	<0.001
Progesterone (ng/ml)	2.6±1.7	3.9±4.0	<0.001
Estradiol (pg/ml)	26.6±11.9	20.9±15.2	<0.001
Testosterone (ng/ml)	6.3±2.1	5.4±1.5	<0.001
**Female subjects**			
** **	**Case (n=138)**	**Control (n=136)**	** **
Age (years)	18.8±0.6	19.6±2.6	<0.001
SEM of right eye (D)	−8.0±1.7	−0.7±0.8	<0.001
SEM of left eye (D)	−7.9±2.1	−0.7±0.7	<0.001
Progesterone (ng/ml)	4.8±4.9	6.6±8.4	0.031
Estradiol (pg/ml)	43.4±26.7	50.2±35.4	0.074
Testosterone (ng/ml)	1.9±1.3	1.2±0.9	<0.001

Data are reported as mean±standard deviation. SE=spherical equivalent; D=diopter.

### Sex steroid levels

The level of progesterone was significantly lower and levels of estradiol and testosterone were significantly higher in the cases than in the controls for male subjects (all p-values <0.001; Table 1). The level of testosterone was also significantly higher in the cases than in the controls for female subjects (p<0.001; Table 1).

### Genotype association

Although no statistically significant deviation from HWE was noted for the controls, the genotypes of all SNPs between the cases and controls were not statistically different (all p-values >0.05; Table 2). Pairwise stratified analyses conducted separately among male and female subjects for all six SNPs (rs743572 [*CYP17A1*], rs6203 [*HSD3B1*], rs1047303 [*HSD3B1*], rs605059 [*HSD17B1*], rs523349 [*SRD5A2*], and rs10046 [*CYP19A1*] were indicated as SNP1–6, respectively) were used to evaluate synergistic modification of association with logistic regression. All the significant results are shown in Table 3. Among male study subjects, SNP6-SNP2 and SNP3-SNP4 combinations showed significant modification of genotypic risks for myopia in both global and specific tests. In the SNP6-AG subgroup, the TC genotype in SNP2 was associated with a high risk of myopia (OR=2.38 [1.17–4.83], p-value=0.017); in the SNP4-GA subgroup, SNP3-AC was associated with a higher risk (OR=4.32 [1.37–13.61], p-value=0.013). Meanwhile, these combinations were related to a protective effect among female subjects (OR=0.40 [0.19–0.81], p-value=0.011 and OR=0.57 [0.21–1.50], p-value=0.253, respectively). Among female subjects, SNP2-TC was associated with a significant risk (OR=2.88 [1.05–7.88], p-value=0.04) in the SNP6-AA subgroup, and SNP2-CC was associated with a protective effect (OR=0.26 [0.08–0.88], p-value=0.03) in the SNP6-AG subgroup; however, there were no significant association among male subjects (OR=0.96 [0.39–2.37], p-value=0.929 and OR=0.42 [0.12–1.46], p-value=0.173, respectively). Table 4 summarizes the best interaction models of seven independent variables (including these six SNPs and sex) in relation to high myopia obtained from the MDR analysis. Consistent with synergistic genotypic association, among all two-factor combinations, the model composed of rs6203 (*HSD3B1*) and rs10046 (*CYP19A1*) was the best attribute for predicting high-myopia risk, though without statistical significance (testing accuracy, 50.6%; p=0.422;cross-validation consistency, 7/10; p=0.429). The best interaction model of all combinations was a three-factor model composed of rs6203 (*HSD3B1*), rs10046 (*CYP19A1*), and sex (testing accuracy, 57.5%; p=0.019; cross-validation consistency, 10/10; p=0.07).

**Table 2:** Association between single nucleotide polymorphisms in sex steroidogenesis enzyme genes and high myopia risk and Hardy–Weinberg equilibrium test.

** **	** **	**High myopia** ** **		**HWE**	**Association results**			
**Gene**	**SNP**	**Case (n=283)**	**Control (n=280)**	**p-value**	**p-value**	**OR (95% CI)**	p-value*	OR (95% CI)*
*CYP17A1*	rs743572	** **	** **	1.000	0.608	** **	0.351	** **
** **	GG	82	92	** **	** **	1.00 (reference)	** **	1.00 (reference)
** **	GA	147	137	** **	** **	1.20 (0.83–1.76)	** **	1.33 (0.90–1.95)
** **	AA	54	51	** **	** **	1.19 (0.73–1.93)	** **	1.26 (0.77–2.06)
*HSD3B1*	rs6203	** **	** **	0.590	0.939	** **	0.887	** **
** **	TT	115	113	** **	** **	1.00 (reference)	** **	1.00 (reference)
** **	TC	138	133	** **	** **	1.02 (0.72–1.45)	** **	1.00 (0.70–1.44)
** **	CC	30	34	** **	** **	0.87 (0.50–1.51)	** **	0.89 (0.50–1.57)
*HSD3B1*	rs1047303	** **	** **	0.620	0.922	** **	0.808	** **
** **	AA	236	239	** **	** **	1.00 (reference)	** **	1.00 (reference)
** **	AC	46	40	** **	** **	1.16 (0.74–1.85)	** **	1.18 (0.73–1.89)
** **	CC	1	1	** **	** **	1.01 (0.06–16.29)	** **	0.82 (0.05–13.34)
*HSD17B1*	rs605059	** **	** **	0.359	0.809	** **	0.821	** **
** **	GG	87	76	** **	** **	1.00 (reference)	** **	1.00 (reference)
** **	GA	140	147	** **	** **	0.83 (0.57–1.22)	** **	0.88 (0.60–1.31)
** **	AA	56	57	** **	** **	0.86 (0.53–1.39)	** **	0.89 (0.55–1.45)
*SRD5A2*	rs523349	** **	** **	0.386	0.776	** **	0.596	** **
** **	GG	97	99	** **	** **	1.00 (reference)	** **	1.00 (reference)
** **	GC	141	129	** **	** **	1.12 (0.77–1.61)	** **	1.17 (0.81–1.71)
** **	CC	45	52	** **	** **	0.88 (0.54–1.44)	** **	0.91 (0.55–1.49)
*CYP19A1*	rs10046	** **	** **	0.983	0.799	** **	0.678	** **
** **	AA	80	87	** **	** **	1.00 (reference)	** **	1.00 (reference)
** **	AG	151	138	** **	** **	1.19 (0.81–1.74)	** **	1.22 (0.83–1.80)
** **	GG	52	55	** **	** **	1.03 (0.63–1.67)	** **	1.22 (0.66–1.84)

SNP=single nucleotide polymorphism; HWE=Hardy–Weinberg equilibrium test (conducted among controls); OR=odds ratio. *Adjusted for sex and age.

**Table 3:** Pairwise stratified analysis of synergistic genotypic association.

**Variable**		**Male**			**Female**		
**SNP6**	**SNP2**	**OR (95% CI)**	**p-value^a^**	**p-value^b^**	**OR (95% CI)**	**p-value^a^**	**p-value^b^**
AA	TT	1.00 (Reference)	** **	0.833	1.00 (Reference)	** **	0.113
** **	TC	0.96 (0.39–2.37)	0.929	** **	2.88 (1.05–7.88)	0.040	** **
** **	CC	1.40 (0.39–5.08)	0.609	** **	1.44 (0.31–6.62)	0.639	** **
AG	TT	1.00 (Reference)	** **	0.007	1.00 (Reference)	** **	0.015
** **	TC	2.38 (1.17–4.83)	0.017	** **	0.40 (0.19–0.81)	0.011	** **
** **	CC	0.42 (0.12–1.46)	0.173	** **	0.26 (0.08–0.88)	0.030	** **
GG	TT	1.00 (Reference)	** **	0.207	1.00 (Reference)	** **	0.829
** **	TC	0.60 (0.18–2.00)	0.406	** **	0.71 (0.23–2.15)	0.541	** **
** **	CC	3.00 (0.48–18.60)	0.238	** **	-	** **	** **
**SNP4**	**SNP3**	**OR (95% CI)**	**p-value^a^**	**p-value^b^**	**OR (95% CI)**	**p-value^a^**	**p-value^b^**
GG	AA	1.00 (Reference)	** **	0.821	1.00 (Reference)	** **	0.609
** **	AC	0.73 (0.27–1.97)	0.821	** **	0.71 (0.19–2.67)	0.609	** **
** **	CC	-	-	** **	-	-	** **
GA	AA	1.00 (Reference)		0.013	1.00 (Reference)		0.253
** **	AC	4.32 (1.37–13.61)	0.013	** **	0.57 (0.21–1.50)	0.253	** **
** **	CC	-	-	** **	-	-	** **
AA	AA	1.00 (Reference)	** **	0.391	1.00 (Reference)	** **	0.835
** **	AC	3.38 (0.59–19.21)	0.391	** **	1.19 (0.24–5.84)	0.835	** **
** **	CC	-	-	** **	-	-	** **

SNP2=rs6203 (*HSD3B1*); SNP3=rs1047303 (*HSD3B1*); SNP4=rs605059 (*HSD17B1*); SNP6=rs10046 (*CYP19A1*). ^a^Specific p-value. ^b^Global p-value.

**Table 4:** Summary of multifactor dimensionality reduction (MDR) analysis.

** **	**Testing results**		**10-fold cross-validation**	
**Factors in the best model**	**Accuracy (%)**	**p-value^a^**	**Consistency**	**p-value^a^**
6	46.0	0.900	3/10	1.000
2, 6	50.6	0.424	7/10	0.429
2, 6. 7	57.5	0.019	10/10	0.070
1, 2, 4, 6	46.6	0.833	5/10	0836

Factors: 1. SNP1: rs743572 (*CYP17A1*); 2. SNP2: rs6203 (*HSD3B1*); 3. SNP3: rs1047303 (*HSD3B1*); 4. SNP4: rs605059 (*HSD17B1*); 5. SNP5: rs523349 (*SRD5A2*); 6. SNP6: rs10046 (*CYP19A1*); 7. Gender. MDR searches through all possible interaction combinations among independent variables (single factor to four-factor interaction models in this study) and reports a combination that provide the best fitness in each model. ^a^Empirical p-values were calculated by permutation analysis.

For all hormones tested, sex as an independent variable was significantly associated with variation, with p<0.001 in all cases (Table 5). The results of ANOVA that included a gene-sex interaction term as an independent variable found that the rs605059 (*HSD17B1*) genotype-sex interaction term was significant (Table 5). In the male subjects, genetic variants of rs605059 (*HSD17B1*) showed between-group differences in estradiol levels (p=0.035; Table 6). In the female subjects, genetic variants of rs605059 (*HSD17B1*) showed between-group differences in testosterone levels (p=0.027; Table 7).

**Table 5:** ANOVA for sex hormone levels among study population, including gene-sex interaction.

** **	**Progesterone**		**Estradiol**		**Testosterone**	
**Variables**	**F**	**p-value**	**F**	**p-value**	**F**	**p-value**
**Univariate**						
Age	0.12	0.727	1.17	0.280	0.10	0.752
Sex	14.64	<0.001	127.09	<0.001	28.80	<0.001
rs743572 (*CYP17A1*)	0.20	0.820	0.93	0.339	1.63	0.198
rs6203 (*HSD3B1*)	0.30	0.743	1.26	0.284	1.59	0.206
rs1047303 (*HSD3B1*)	0.00	0.996	0.77	0.461	0.67	0.512
rs605059 (*HSD17B1*)	0.23	0.796	0.85	0.428	0.57	0.566
rs523349 (*SRD5A2*)	0.30	0.741	0.66	0.517	0.51	0.603
rs10046 (*CYP19A1*)	0.25	0.783	0.24	0.786	0.23	0.795
**Interaction**						
rs743572 (*CYP17A1*)* Sex	0.55	0.580	1.62	0.199	1.25	0.782
rs6203 (*HSD3B1*)* Sex	0.16	0.856	0.60	0.547	0.64	0.526
rs1047303 (*HSD3B1*)* Sex	0.35	0.555	1.01	0.315	1.08	0.298
rs605059 (*HSD17B1*)* Sex	1.50	0.225	3.38	0.035	3.09	0.046
rs523349 (*SRD5A2*)* Sex	0.10	0.904	0.57	0.568	0.47	0.624
rs10046 (*CYP19A1*)* Sex	0.45	0.640	1.14	0.320	1.14	0.320

F=The effect of an independent variable on hormone levels after adjustment for all other independent variables.

**Table 6:** Genotypes and sex hormone levels among male subjects

** **	**Progesterone (ng/ml)**		**Estradiol (pg/ml)**		**Testosterone (ng/ml)**	
**Study population**	**Mean**	**Standard deviation**	**Mean**	**Standard deviation**	**Mean**	**Standard deviation**
rs743572** (*CYP17A1*)**						
GG (n=76)	3.12	2.01	24.27	14.44	6.10	1.80
GA (n=156)	3.45	3.88	24.34	14.19	5.75	1.87
AA (n=57)	2.99	1.69	21.55	12.48	5.75	2.16
p-value	0.494		0.358		0.438	
rs6203** (*HSD3B1*)**						
TT (n=120)	3.48	4.19	23.49	14.38	5.93	1.95
TC (n=133)	3.04	2.09	24.03	13.93	5.74	1.89
CC (n=36)	3.44	1.92	23.76	12.72	5.94	1.86
p-value	0.483		0.967		0.684	
rs1047303** (*HSD3B1*)**						
AA (n=239)	3.37	3.36	23.86	14.18	5.89	1.84
AC (n=48)	2.75	1.49	23.00	12.86	5.68	2.24
CC (n=2)	3.97	3.01	32.61	6.85	4.34	0.54
p-value	0.454		0.653		0.414	
rs605059** (*HSD17B1*)**						
GG (n=83)	2.77	1.50	20.50	11.08	5.67	1.72
GA (n=151)	3.42	2.88	24.80	14.21	5.92	2.09
AA (n=55)	3.60	5.00	25.88	16.24	5.91	1.64
p-value	0.214		0.035		0.600	
rs523349** (*SRD5A2*)**						
GG (n=110)	2.92	1.88	21.63	11.15	5.91	1.93
GC (n=138)	3.58	4.12	25.63	15.84	5.84	1.96
CC (n=41)	3.17	1.38	23.31	13.21	4.70	1.71
p-value	0.217		0.066		0.866	
rs10046** (*CYP19A1*)**						
AA (n=91)	2.86	1.42	21.35	11.51	5.88	1.88
AG (n=146)	3.52	3.83	25.50	14.50	5.85	1.90
GG (n=52)	3.29	3.07	23.15	15.64	5.77	2.00
p-value	0.249		0.064		0.960	

p-values were adjusted by age.

**Table 7:** Genotypes and sex hormone levels among female subjects.

** **	**Progesterone (ng/ml)**		**Estradiol (pg/ml)**		**Testosterone (ng/ml)**	
**Study population**	**Mean**	**Standard Deviation**	**Mean**	**Standard Deviation**	**Mean**	**Standard Deviation**
rs743572** (*CYP17A1*)**						
GG (n=98)	5.33	7.13	42.98	28.69	1.41	0.86
GA (n=128)	5.98	7.30	50.53	34.26	1.64	1.35
AA (n=48)	5.71	5.22	44.39	28.08	1.67	1.07
p-value	0.780		0.171		0.257	
rs6203** (*HSD3B1*)**						
TT (n=108)	5.70	7.40	44.16	30.31	1.71	1.38
TC (n=138)	5.59	6.17	48.74	33.11	1.48	0.98
CC (n=28)	6.26	8.43	46.95	27.11	1.41	0.85
p-value	0.799		0.386		0.361	
rs1047303** (*HSD3B1*)**						
AA (n=236)	5.66	6.80	47.27	30.56	1.58	1.14
AC (n=38)	5.97	7.63	43.57	36.64	1.43	1.21
p-value	0.666		0.569		0.484	
rs605059** (*HSD17B1*)**						
GG (n=80)	6.34	6.65	50.64	34.79	1.83	1.44
GA (n=136)	5.54	7.27	47.43	31.87	1.44	0.94
AA (n=58)	5.18	6.42	39.82	23.97	1.48	1.10
p-value	0.425		0.114		0.027	
rs523349** (*SRD5A2*)**						
GG (n=86)	5.53	7.16	48.03	30.78	1.52	1.02
GC (n=132)	5.94	7.48	47.59	34.06	1.61	1.35
CC (n=56)	5.41	4.90	42.83	25.56	1.49	0.78
p-value	0.977		0.612		0.846	
rs10046** (*CYP19A1*)**						
AA (n=76)	6.13	8.02	50.83	36.93	1.50	1.00
AG (n=143)	5.37	6.25	45.34	29.38	1.60	1.16
GG (n=55)	5.97	6.95	44.79	28.19	1.56	1.32
p-value	0.843		0.552		0.546	

p-values were adjusted by age.

Our results reveal that synergistic interaction of rs6203 (*HSD3B1*), rs10046 (*CYP19A1*), and sex may confer susceptibility to high myopia; testosterone levels for either sex correlate significantly with high myopia; and rs605059 (*HSD17B1*) interacting with sex-related factors may be associated with circulating sex hormone levels in high myopia.

## Discussion

To our knowledge, this is the first study that describes the relationship between polymorphisms in steroidogenesis enzyme genes and high-myopia risk and their correlations with serum sex hormone levels. The principle findings of this study are the identification of (1) the combination of rs6203 (*HSD3B1*), rs10046 (*CYP19A1*), and sex may confer susceptibility to high myopia; (2) significant differences in all progesterone, estradiol, and testosterone levels between high-myopia cases and controls in males and significant differences in testosterone levels between high-myopia cases and controls in females; (3) an association between rs605059 (*HSD17B1*) and serum estradiol levels in males and serum testosterone levels in females; and (4) sexual dimorphism of these associations, which suggests a sex-gene interaction.

SNP information was obtained from reviewing papers [48-59] and from GenBank. We validated the presence of six common SNPs in our study. A comparison with four populations from the HapMap database (743572, 6203, 1047303, 605059, 523349, and 10046; accessed October 1, 2009) revealed significant differences in allele frequencies between Taiwanese controls and both Utah, United States, residents with European ancestry (CEU) and the Yoruba population in Ibadan, Nigeria (YRI; p<0.001; Table 8). The between-population differences noted here may help explain the well known differences in serum hormone levels and the prevalence of hormone-related diseases between Asian and Caucasian populations [64,65]. A lack of deviation from HWE among controls (Table 2) indicated that population stratification or genotyping errors are not likely. As the first study investigating an association between polymorphisms in steroidogenesis enzyme genes and high-myopia risk, this study did not observe statistically significant associations in any single variant. However, it is conceivable that particular combinations of multiple variants may confer an enhanced susceptibility to high myopia. Both stratified analysis of genotypic association (parametric) and MDR analysis (non-parametric) confirmed a synergistic interaction among rs6203 (*HSD3B1*), rs10046 (*CYP19A1*), and sex. The *HSD3B* gene family has two genes and five pseudogenes, all of which map to chromosome 1p13 [66-68]. The *HSD3B1* gene encodes the type I enzyme, which is exclusively expressed in the placenta and peripheral tissues, such as prostate, breast, and skin. The *HSD3B2* gene encodes the type II enzyme, which is predominantly expressed in classical steroidogenic tissues, namely the adrenals, testis, and ovary [68-71]. 3β-hydrosteroid dehydrogenases (HSD3B1) catalyze several reactions in the androgen pathway (Figure 1), leading to production of androstenedione and testosterone. They also catalyze the production of progesterone from pregnenolone. Cytochrome P-450 19A1 (*CYP19A1*), located on chromosome 15, codes for aromatase, which is involved in the rate-limiting step in estrogen production. It is mainly expressed in the ovarian granulose cells; however, aromatase is also expressed in several other tissues, including fat, placenta, breast, central nervous system, skin, and bone. Rosmond et al. [51] proposed that genetic variation in *HSD3B1* might lead to an elevation in plasma aldosterone (one of the mineralocorticoids, which are downstream products of progestogens), with a resultant increase in intravascular volume and hypertension, and they did prove the rs6203 silent substitution in exon 4 of *HSD3B1* was associated with elevated blood pressure in a population of Swedish men. Thus, it is possible that genetic variations in these steroidogenesis enzyme genes might also influence sex steroids and the pathogenesis of steroid-related diseases. Our results also support the associations between genetic variations of steroidogenesis enzyme genes and high myopia.

**Table 8:** Comparison of allele frequencies between Taiwanese and other populations.

** **		**Study population**				
** Gene**	**Allele**	**Taiwan (n=280)**	**CEU (n=116)**	**YRI (n=118)**	**HCB (n=90)**	**JPT (n=88)**
rs743572 (*CYP17A1*)	A	0.427	0.603	0.729	0.456	0.625
** **	G	0.573	0.397	0.271	0.544	0.375
** **	p-value	** **	<0.001	<0.001	0.498	<0.001
** **	** **	Taiwan (n=280)	CEU (n=120)	YRI (n=120)	HCB (n=90)	JPT (n=88)
rs6203 (*HSD3B1*)	C	0.359	0.608	1.000	0.322	0.375
** **	T	0.641	0.392	0.000	0.678	0.625
** **	p-value	** **	<0.001	<0001	0.369	0.699
** **	** **	Taiwan (n=280)	CEU (n=120)	YRI (n=120)	HCB (n=90)	JPT (n=88)
rs1047303 (*HSD3B1*)	A	0.925	0.992	0.992	0.900	0.898
** **	C	0.075	0.008	0.008	0.100	0.102
** **	p-value	** **	<0.001	<0.001	0.285	0.249
** **	** **	Taiwan (n=280)	CEPH (n=184)	YRI (NA)	HCB (NA)	JPT (NA)
rs605059 (*HSD17B1*)	A	0.466	0.730	** **	** **	** **
** **	G	0.534	0.270	** **	** **	** **
** **	p-value	** **	<0.001	** **	** **	** **
** **	** **	Taiwan (n=280)	CEU (n=120)	YRI (n=120)	HCB (n=90)	JPT (n=86)
rs523349 (*SRD5A2*)	C	0.416	0.808	0.775	0.522	0.640
** **	G	0.584	0.192	0.225	0.478	0.360
	p-value	** **	<0.001	<0.001	0.013	<0.001
** **	** **	Taiwan (n=280)	CEU (n=110)	YRI (n=120)	HCB (n=90)	JPT (n=90)
rs10046 (*CYP19A1*)	C	0.557	0.445	0.825	0.411	0.578
** **	T	0.443	0.555	0.175	0.589	0.422
** **	p-value	** **	0.005	<0.001	<0.001	0.627

CEU: Utah residents with Northern and Western European ancestry from the CEPH collection; YRI: Yoruba in Ibadan, Nigeria; HCB: Han Chinese in Beijing, China; JPT: Japanese in Tokyo, Japan; CEPH: Centre d’Etude du Polymorphisme Human pedigrees; NA: not available. p-values were calculated by pairwise comparisons of allele frequencies between the Taiwanese population and other populations.

This current study showed that high-myopia cases have significantly higher levels of testosterone in both sexes, suggesting that higher testosterone levels for either sex are associated with an increased risk of high myopia. Additionally, in males, serum levels of estradiol are significantly higher and serum levels of progesterone are significantly lower in the high-myopia group. The possibility that steroid levels are associated with an increased rate of axial elongation requires further elucidation by a longitudinal study. Previously, Balacco et al. [72] reported higher levels of steroids (testosterone) in high-myopia cases (in excess of −10 D). Plasma cortisol levels have been shown to be lower in myopia cases than in normal subjects [73], although the results were not highly significant and the study size was small. In another case-control study, no significant differences were noted in serum levels of cortisol, testosterone, or estradiol between high myopes and non-myopes (p>0.1) [74], but the study size was also small. The number of subjects for our study is relatively large, and the results are significant (all p-values <0.001). Modulated by retinoscleral signals, the scleral remodeling mechanism is intrinsic to myopia [13]. The existence of sex steroid receptors in the retina [24,26,28] makes an effect of sex hormones on retinoscleral signals plausible.

Estrogen has been shown to upregulate MMP-2 and/or MMP-9 in human ocular cells [26,45], and MMP-2 upregulation was suggested as part of the scleral remodeling process in myopia development [17,23]. The finding of higher estradiol levels in male high-myopia cases is consistent with our hypothesis that differential sex hormone levels may play a role in myopia pathogenesis by regulating MMP-2.

Because steroidogenesis enzymes catalyze the biosynthesis of sex hormones, we examined whether polymorphisms of these enzymes influence the serum levels of sex hormones. Our data suggested that for males, rs605059 (*HSD17B1*) A allele is associated with a significant increase in the level of estradiol and a non-significant increase in the level of testosterone; for females, it is associated with a non-significant decrease in the level of estradiol and a significant decrease in the level of testosterone. This sexual dimorphism could be the result of a sex-gene interaction we demonstrated by ANOVA analysis (Table 5), although these results of female subjects should be interpreted with caution because of limitations to the present association study. The effect of polymorphisms in estrogen-metabolizing genes on circulating estrogen levels would be most pronounced in women with no other sources of estrogens because peripheral conversion of androgens in the adipose tissue or hormone replacement therapy (HRT) may mask the genetic effect. In addition, fluctuations in progesterone and estradiol levels during the menstrual cycle or pregnancy may obscure a modest genetic effect. Hence, a future study with a larger sample size and more considerable lifestyle information will help match controls to cases regarding various confounders—such as age, body mass index (BMI), pregnancy or menopausal status, HRT usage, day of period, time of day, and fasting status at blood draw—and determine these associations in females. Evidence proves that sexual dimorphism of the muscarinic system exists in the human brain and bladder [75-79]. In rat models, sex steroids selectively modulate muscarinic receptors in certain brain tissues (e.g., hypothalamus, adenohypophysis, superior cervical ganglia, or anterior pituitary gland) in a region-specific manner but not in the retina [80-85]. The muscarinic receptors and hormone receptors might be in different steps of a common pathway in myopia. Previous studies that have examined the rs605059 (*HSD17B1*) variant in relation to estrogens found no association [86-88]. The enzymes 17 beta-hydroxysteroid dehydrogenase 1 (*HSD17B1*) and 17 beta-hydroxysteroid dehydrogenase 2 (*HSD17B2*) are responsible for the reduction and oxidation of estrogens to more- and less-active forms, respectively. *HSD17B1* is responsible for the reduction of estrone to estradiol, the more bioactive estrogen; *HSD17B2* is responsible for the oxidation of estradiol to estrone. In addition to activating estrone to estradiol, human *HSD17B1* enhances androgen activity in vivo [89]. The functional significance of rs605059 (*HSD17B1*) is unclear, but it appears to have little effect on the catalytic or immunological properties of this enzyme [90] and is indicated by sequence homology analysis (though with low confidence) that it is unlikely to affect function [91].

We observed associations between certain combinations of polymorphisms at steroidogenesis enzyme genes (rs6203 [*HSD3B1*], rs10046 [*CYP19A1*], and sex) and high-myopia risk; meanwhile, there is evidence of correlations between rs605059 (*HSD17B1*)–sex interaction and sex hormone levels. Sex hormones may play only a modulating rather than mediating role in the formation of high myopia; thus, the genotype associations with hormone levels and high-myopia traits are not parallel, and the small genetic effect of genotype-disease association cannot be detected unless a stratified synergistic association analysis is applied. The complex underlying genetic mechanism, especially in complex diseases, warrants consideration of environment-gene and gene-gene interactions. Our study did clearly reveal that a sex-gene interaction plays a significant role in sex hormone levels and high-myopia traits as well. A larger sample size and more comprehensive confounder controlling will further help elucidate the associations.

In conclusion, these findings reflect the unique genetic characteristics of the Han Chinese population. We found significant correlations between sex steroid levels and high myopia, and the synergistic interaction of sex and steroidogenesis enzyme genes might confer susceptibility to high myopia and influence sex steroid levels. Further studies are required to confirm this relationship. Sex-gene interaction is crucial for understanding sex hormone metabolism and possible high-myopia risk.
